# RETRACTION: Synthesis and Cardiovascular Protective Effects of Quercetin 7‐O‐Sialic Acid

**DOI:** 10.1111/jcmm.70467

**Published:** 2025-02-26

## Abstract

**RETRACTION:** H. Tian, Q. Liu, S. Qin, C. Zong, Y. Zhang, S. Yao, N. Yang, T. Guan, and S. Guo, “Synthesis and Cardiovascular Protective Effects of Quercetin 7‐O‐Sialic Acid,” *Journal of Cellular and Molecular Medicine* 21, no. 1 (2017): 107–120, https://doi.org/10.1111/jcmm.12943.

The above article, published online on 11 August 2016, in Wiley Online Library (wileyonlinelibrary.com), has been retracted by agreement between the authors, the journal Editor‐in‐Chief, Stefan N. Constantinescu, the Foundation for Cellular and Molecular Medicine, and John Wiley and Sons Ltd. Following publication, concerns were raised by third parties that Figures 3 and 5 contained duplicated images. The authors acknowledged the duplications but were unable to provide the uncropped western blot raw data. The retraction has been agreed upon because of the duplication concerns, affecting the interpretation of the data and results presented.

**RETRACTION:** H. Tian, Q. Liu, S. Qin, C. Zong, Y. Zhang, S. Yao, N. Yang, T. Guan, and S. Guo, “Synthesis and Cardiovascular Protective Effects of Quercetin 7‐O‐Sialic Acid,” *Journal of Cellular and Molecular Medicine* 21, no. 1 (2017): 107–120, https://doi.org/10.1111/jcmm.12943.

The above article, published online on 11 August 2016, in Wiley Online Library (wileyonlinelibrary.com), has been retracted by agreement between the authors, the journal Editor‐in‐Chief, Stefan N. Constantinescu, the Foundation for Cellular and Molecular Medicine, and John Wiley and Sons Ltd. Following publication, concerns were raised by third parties that Figures 3 and 5 contained duplicated images. The authors acknowledged the duplications but were unable to provide the uncropped western blot raw data. The retraction has been agreed upon because of the duplication concerns, affecting the interpretation of the data and results presented.

